# Prevalence and Socio-Demographic Determinants of Overweight and Obesity in a Nigerian Population

**DOI:** 10.2188/jea.JE20140099

**Published:** 2015-07-05

**Authors:** Fatai A. Maruf, Nwannedimma V. Udoji

**Affiliations:** Department of Medical Rehabilitation, Faculty of Health Sciences and Technology, Nnamdi Azikiwe University, Nnewi, Anambra State, Nigeria

**Keywords:** determinants, obesity, overweight, prevalence, developing country

## Abstract

**Objective:**

This survey explored prevalence of overweight and obesity and their associations with socio-demographic variables in a Nigerian population.

**Methods:**

This cross-sectional survey involved 1521 adults in Nnewi. Age, sex, educational and occupational status, and BMI were recorded.

**Results:**

Prevalence of overweight was higher in males (32.3%; 95% CI, 29.5%–35.2%) than in females (29.8%; 95% CI, 26.8%–33.0%); the reverse was the case for prevalence of obesity (19.6%; 95% CI, 17.3%–22.2% in males and 36.0%; 95% CI, 32.8%–39.4% in females). Higher odds ratios (ORs) for overweight and obesity were observed in participants aged 41–60 years (OR 2.03; 95% CI, 1.57–2.61 for overweight and OR 4.29; 95% CI, 3.25–5.67 for obesity) and those >60 years (OR 1.72; 95% CI, 1.21–2.43 for overweight and OR 4.21; 95% CI, 2.86–6.19 for obesity) compared to those aged 18–40 years. Female sex was associated with higher ORs for overweight (OR 1.20; 95% CI, 0.96–1.51) and obesity (OR 2.21; 95% CI, 1.73–2.83). Participants with secondary education had marginally higher ORs for overweight (OR 1.15; 95% CI, 0.88–1.51) and obesity (OR 1.17; 95% CI, 0.86–1.59) than those with tertiary education, and so were those with primary education for obesity (OR 1.19; 95% CI, 0.74–1.89) but higher OR for overweight (OR 1.44; 95% CI, 0.98–2.13). Unskilled participants had about the same OR for overweight and obesity as professionals, and while skilled participants had about the same OR for overweight as professionals, their OR for obesity (OR 1.27; 95% CI, 0.67–2.43) was fairly higher than that for professionals.

**Conclusions:**

Prevalence of overweight is higher in males than in females, but the reverse is the case for prevalence of obesity. Older age and female sex are associated with increased risk of overweight and obesity, while working at a skilled occupation is associated with obesity, and tertiary educational attainment is associated with overweight.

## INTRODUCTION

Overweight and obesity have reached epidemic proportions globally, with more than a billion overweight adults, and are major contributors to the global burden of chronic disease and disability.^1^ Overweight and obesity are an important public health issue and are among the leading health indicators in the United States.^1^ In the developing world, which was previously unaffected, prevalence of obesity and obesity-related diseases has increased,^2^ and affects physical and social functioning and quality of life.^3^

Increases in prevalence of overweight and obesity among both adults and children have been observed in many countries worldwide.^5^^,^^6^ As incomes rise and populations become more urban, diets high in complex carbohydrates give way to more varied diets with a higher proportion of saturated fats and sugars.^1^ Further, large shifts towards less physically-demanding work, such as increasing use of automated transport, use of technology in the home, and more passive leisure pursuits, have been observed worldwide.^1^ These lifestyles promote excessive caloric intake and sedentary patterns that induce a positive energy balance leading to weight gain.^7^

Socioeconomic status (SES) is the ranking of individuals within complex societies.^8^ In urban Cameroon, higher SES is associated with obesity.^9^ In Thailand, however, an association exists between higher SES and obesity in males, but the association is reversed in females.^10^ Obesity/overweight is associated with high education and income levels in the female Malay population living in Singapore, whereas the association in males is inverse.^11^ Among the female Chinese population in Singapore, low income, low education, and indigent housing status are associated with overweight/obesity.^12^ Associations between SES and overweight/obesity clearly differ from country to country.

Studies on overweight and obesity in Nigerian populations abound.^2^^,^^13^^–^^20^ However, of all these studies, only Desalu et al,^13^ Mbada et al,^16^ and Olatubosun et al^18^ examined prevalence of overweight and obesity in relation to SES. Olatubosun et al reported that overweight and obesity were associated with higher SES in urban Nigerian adults, whereas Mbada et al reported inverse associations between obesity and SES in male and female semi-urban Nigerian adults.^16^^,^^18^ On the contrary, Desalu et al reported no association between SES and obesity.^13^ In fact, two recent reviews of studies on obesity in relation to SES in developing countries did not include any study from Nigeria, despite it being a highly populous developing country in Africa.^21^^,^^22^ Thus, considering the mixed and sparse nature of findings of the Nigerian studies on SES in relation to overweight/obesity, the present study explored the prevalence of overweight and obesity and the associations of gender, age, and socioeconomic indices with overweight/obesity in adult residents of Nnewi, south-eastern Nigeria.

Nnewi is a sub-urban town in southeast Nigeria located some 15 miles from Onitsha, the largest commercial city in south-eastern Nigeria.^23^ It is a commercial and industrial centre with a lot of traders and manufacturers of diverse items, as well as importers of goods (mainly motorcycles and their spare parts) from overseas countries, such as China. About 25 medium- to large-sized factories, including a motor vehicle assembly factory, are fully in operation in Nnewi. Nnewi, together with two other commercial cities in the region, form part of south-eastern Nigeria’s “industrial axis”.^23^ In fact, Nnewi is popularly dubbed “Taiwan of Africa”, while some others refer to it as Nigeria’s “Japan”.^23^ These commercial activities attract people from various parts of Nigeria.^24^ With this commercial influence, although education is free, enrolment of male youngsters in schools, especially at the secondary school level, is low compared with that of females. This low enrolment may be attributed to an increasing number of boys entering the labour market as sales boys or serving as trainee artisans, under a master, for some years.^24^ These youngsters justify their options with the saying that the educated ones have nothing to show for it in terms of money.^25^ However, some of these boys are forced into business and still yearn for formal education, especially at the post-secondary level, but cannot gain the desired access to it.^25^ Therefore, a large proportion of the male population in Nnewi engage in buying and selling and are proud to be addressed as importers and exporters of goods.

## METHODS

This cross-sectional survey was part of a larger study on socio-demographic influences on body weight, perceived body weight, and body image. Participants and procedures for data collection have been described elsewhere.^26^ Briefly, 1521 residents of Nnewi (775 males and 746 females) aged 18 to 85 years who had lived in the area for at least 2 years were involved in this study. As of 2006, Nnewi had a population of 391 227 with a land area of 1076.9 square miles according to the Nigerian census.^24^ There are four quarters in Nnewi: Otolo, Umudim, Uruagu, and Nnewi-ichi. Three churches were randomly selected from the list of churches from each of the quarters, giving a total of 12 randomly selected churches. As an overwhelming majority of Nnewi residents are of Christian faith, churches were used as the unit of randomization.^23^ A minimum sample size of 1388 participants was estimated. However, at the end of data collection, 1521 respondents were included in data analysis. The sample size was estimated as described elsewhere.^26^ The participants gave their informed verbal consent to participate in this study. Exclusion criteria included being pregnant, having a deformity (such as polio paralysis) that may make height measurement unreliable, and lower limb amputation that may make weight measurement unreliable due to problems with weight distribution on the scale. The Ethics Committee of the Nnamdi Azikiwe University Teaching Hospital, Nnewi approved this study. Age at the last birthday of participants and sex of participants were recorded. We also took anthropometric measurements and assessed SES using a questionnaire.

### Anthropometric measurements

Height was measured to the nearest 1 cm using a height meter (Model 786; Seca, Hamburg, Germany). Participants were barefoot and stood on the platform of the height meter while the height was read from the meter at the level of the vertex of the head. Weight was measured to the nearest 1 kg using a weighing scale (Model BR9011, 120 × 0.01 kg; Hana, Hengshui, China). Participants were weighed barefoot and wearing light clothing. Before the participants got on the weighing scale, the pointer of the meter was set at the zero point. The weight was read off the meter by bending over it. Body mass index (BMI) of the participants was calculated from their respective height and weight as weight (kg) divided by height (m) squared. Based on BMI, participants were categorised as underweight, normal weight, overweight, or obese using the World Health Organization criteria.^27^

### Socio-economic assessment

SES was examined using occupational and educational indices. Information on the participants’ occupational status and highest educational attainment was collected from the participants using a questionnaire. The occupations of participants were ranked as social class I (SC I; professional), social class II (SC II; managerial and technical), social class III (SC III; skilled-manual and non-manual), social class IV (SC IV; partly skilled), and social class V (SC V; unskilled).^28^ A housewife was classified by the occupation of her main breadwinner in the past year. Unemployed individuals were classified by their former job if they quit the job not more than 6 months ago; otherwise, they were classified as unskilled. Retired individuals were classified by the job from which they retired. The participants’ highest educational attainment was ranked as pre-primary (0), primary (1), lower secondary (2), upper secondary (3), post-secondary non-tertiary (4), first stage of tertiary education (5), and second stage of tertiary education (6).^29^

### Data analysis

The data were summarised using mean and standard deviation from complex samples. Complex samples frequency was used to present the distribution of participants across socioeconomic categories and to determine prevalence of overweight and obesity of participants. Complex samples multinomial logistic regression was used to determine risk estimates for overweight and obesity, with age group, gender, highest educational attainment, and occupational status as factors. Adjustments were made for age, gender, occupational status, and educational attainment. Reference categories were the 18–40 years age group for age, male for sex, tertiary education for educational attainment, and professional occupation for occupational status. The reference BMI category was normal weight. Statistical level of error was set at 0.05. The Statistical Package of Social Sciences (SPSS) version 21 (IBM, Armonk, NY, USA) was used for data analysis.

## RESULTS

The mean estimated age and BMI of participants in this study, as well as education and occupation, are shown in Table 1. Males comprised 55.6% (53.4%–57.9%) of participants. The majority of participant were aged ≤40 years, had secondary education, and worked in unskilled occupations. The overall prevalences of overweight and obesity were 31.2% (95% confidence interval [CI], 29.1%–33.4%) and 26.9% (95% CI, 24.9%–29.0%), respectively. The prevalence of overweight tended to be higher in males (32.3%; 95% CI, 29.5%–35.2%) than in females (29.8%; 95% CI, 26.8%–33.0%), whereas the reverse was the case for prevalence of obesity (19.6%; 95% CI, 17.3%–22.2% in males and 36.0%; 95% CI, 32.8%–39.4% for females).

**Table 1.** Socio-demographic characteristics of participants and distributions of variable in the sample by sex

	Male	Female	Total
	Mean Estimate (SE)	Mean Estimate (SE)	Mean Estimate (SE)
Age (years)	42.41 (0.46)	45.94 (0.44)	43.98 (0.33)
BMI (kg/m^2^)	25.94 (0.17)	28.31 (0.21)	26.99 (0.14)

	% of total (95% CI)	% of total (95% CI)	% of total (95% CI)

Age (years)			
18–40	54.9 (51.8–57.9)	42.3 (38.9–45.7)	49.3 (47.0–51.6)
41–60	31.6 (28.8–34.5)	41.7 (38.4–45.1)	36.1 (33.9–38.3)
>60	13.5 (11.6–15.8)	16.0 (13.7–18.7)	14.6 (13.1–16.3)
Body weight			
Underweight	3.0 (2.1–4.2)	1.4 (0.8–2.5)	2.3 (1.7–3.1)
Normal weight	45.1 (42.1–48.1)	32.8 (29.7–36.0)	39.6 (37.4–41.9)
Overweight	32.3 (29.5–35.2)	29.8 (26.8–33.0)	31.2 (29.1–33.4)
Obese	19.6 (17.3–22.2)	36.0 (32.8–39.4)	26.9 (24.9–29.0)
Education			
None/primary	11.8 (10.0–13.9)	14.5 (12.3–17.1)	13.0 (11.4–14.6)
Secondary	60.6 (57.6–63.6)	57.8 (54.4–61.1)	59.4 (57.1–61.6)
Tertiary	27.6 (24.9–30.4)	27.7 (24.8–30.8)	27.6 (25.6–29.7)
Occupation			
Professional	4.8 (3.7–6.3)	4.1 (3.0–5.6)	4.5 (3.7–5.5)
Skilled	26.6 (24.0–29.5)	39.0 (35.7–42.4)	32.1 (30.0–34.3)
Unskilled	68.5 (65.6–71.3)	56.9 (53.5–60.3)	63.4 (61.1–65.5)

CI, confidence interval; SE, standard error.

The prevalence of overweight tended to be highest in the 41–60 years age group (33.3%; 95% CI, 29.7%–37.0%), and the prevalence of obesity tended to be highest in the age group >60 years (38.5%; 95% CI, 32.9%–44.4%) (Table 2). The prevalence of overweight and obesity tended to decrease steadily across educational groups from uneducated/primary education participants. Prevalence of overweight tended to be highest among the participants with unskilled occupations (31.9%; 95% CI, 29.3%–34.7%), but prevalence of obesity tended to be highest among those with skilled occupations (31.0%; 95% CI, 27.4%–34.9%).

**Table 2.** Socio-demographic characteristics of participants and distributions of variable in the sample across BMI categories

	Underweight	Normal weight	Overweight	Obese
	% of total (95% CI)	% of total (95% CI)	% of total (95% CI)	% of total (95% CI)
Age (years)				
18–40	3.1 (2.1–4.4)	51.9 (48.7–55.1)	29.7 (26.9–32.8)	15.3 (13.0–17.8)
41–60	1.4 (0.7–2.6)	27.3 (24.0–30.8)	33.3 (29.7–37.0)	38.1 (34.4–41.9)
>60	1.9 (0.8–4.3)	28.6 (23.6–34.2)	31.1 (25.9–36.8)	38.5 (32.9–44.4)
Gender				
Male	3.0 (2.1–4.2)	45.1 (42.1–48.1)	32.3 (29.5–35.2)	19.6 (17.3–22.2)
Female	1.4 (0.8–2.5)	32.8 (29.7–36.0)	29.8 (26.8–33.0)	36.0 (32.8–39.4)
Education				
None/primary	—	32.4 (26.7–38.5)	35.7 (29.8–42.0)	32.0 (26.4–38.2)
Secondary	2.7 (0.5)	38.3 (35.5–41.2)	31.4 (28.7–34.2)	27.7 (25.1–30.4)
Tertiary	2.6 (0.7)	46.0 (41.7–50.3)	28.8 (25.0–32.8)	22.7 (19.2–26.6)
Occupation				
Professional	—	47.9 (37.9–58.1)	31.0 (22.2–41.4)	21.1 (13.4–31.6)
Skilled	2.4 (1.4–4.0)	36.8 (33.0–40.8)	29.8 (26.2–33.6)	31.0 (27.4–34.9)
Unskilled	2.4 (1.7–3.4)	40.5 (37.7–43.3)	31.9 (29.3–34.7)	25.2 (22.8–27.8)

CI, confidence interval.

The participants aged 41–60 years had higher odds ratios (ORs) for overweight (OR 2.03; 95% CI, 1.57–2.61) and obesity (OR 4.29; 95% CI, 3.25–5.67) relative to normal weight than those aged 18–40 years, as did those above 60 years of age (overweight OR 1.72; 95% CI, 1.21 to 2.43; obesity OR 4.21; 95% CI, 2.86–6.19) (Table 3). Similarly, females had higher ORs for overweight (OR 1.20; 95% CI, 0.96–1.51) and obesity (OR 2.21; 95% CI, 1.73–2.83) than males.

**Table 3.** Odds ratio for overweight and obesity from demographic and socioeconomic factors

	Univariate		Multivariate	
	
	Underweight	Normal weight	Overweight	Obese
	OR (95% CI)	OR (95% CI)	OR (95% CI)	OR (95% CI)
Age (years)				
18–40^a^	1.0	1.0	1.0	1.0
41–60	2.13 (1.60–2.83)	4.75 (3.47–6.49)	2.03 (1.57–2.61)^b^	4.29 (3.25–5.67)^b^
>60	1.89 (1.28–2.81)	4.59 (3.47–6.49)	1.72 (1.21–2.43)^b^	4.21 (2.86–6.19)^b^
Gender				
Male^a^	1.0	1.0	1.0	1.0
Female	1.27 (0.98–1.64)	2.52 (1.92–3.31)	1.20 (0.96–1.51)^c^	2.21 (1.73–2.83)^c^
Education				
None/primary^a^	1.0	1.0	1.0	1.0
Secondary	1.31 (0.99–1.74)	1.46 (1.07–2.00)	1.15 (0.88–1.51)^d^	1.17 (0.86–1.59)^d^
Tertiary	1.76 (1.15–2.69)	2.00 (1.28–3.13)	1.44 (0.98–2.13)^d^	1.19 (0.74–1.89)^d^
Occupation				
Professional^a^	1.0	1.0	1.0	1.0
Skilled	1.25 (0.69–2.26)	1.91 (0.95–3.85)	0.99 (0.58–1.70)^e^	1.27 (0.67–2.43)^e^
Unskilled	1.22 (0.69–2.15)	1.42 (0.72–2.79)	0.99 (0.58–1.68)^e^	1.02 (0.54–1.97)^e^

CI, confidence interval.

^a^Reference category.

^b^Adjusted for gender, occupational status, and educational attainment.

^c^Adjusted for age, occupational status, and educational attainment.

^d^Adjusted for gender, occupational status, and age.

^e^Adjusted for gender, age, and educational attainment.

Participants with primary (OR 1.44; 95% CI, 0.98–2.13) and secondary (OR 1.15; 95% CI, 0.88–1.51) education had fairly higher and marginally higher ORs for overweight, respectively, than those with tertiary education (Table 3). However, participants with primary (OR 1.19; 95% CI, 0.74–1.89) and secondary (OR 1.17; 95% CI, 0.86–1.59) education had marginally higher OR for obesity than those with tertiary education. The participants in skilled (OR 0.99; 95% CI, 0.58–1.70) and unskilled (OR 0.99; 95% CI, 0.58–1.68) occupations had about the same OR for overweight as those in professional occupations. However, as those in skilled occupations had fairly higher OR for obesity (OR 1.27; 95% CI, 0.67–2.43) than those who were professionals, those in unskilled occupations had about the same OR for obesity as those with professional occupations (OR 1.02; 95% CI, 0.54–1.97).

## DISCUSSION

This study examined the prevalences of overweight and obesity and their associations with socio-demographic variables in a south-eastern Nigerian population. The overall prevalences of overweight and obesity were 31.2% and 26.9%, respectively. The prevalence of overweight in males and females was 32.3% and 29.8%, respectively; respective prevalence of obesity was 19.6% and 36.0%. Participants aged 41–60 years had a higher risk of having larger BMI than those aged 18–40 years, and those aged >60 years had an even higher risk. Females had a higher risk for both overweight and obesity than males. However, while tertiary educational attainment was associated with overweight, skilled occupational status was associated with obesity.

The prevalence of overweight observed in this study falls within the range (20.3% to 35.1%) reported in a review of Nigerian studies on overweight, while the prevalence of obesity is higher than the range reported (8.1% to 22.2%).^30^ Further, the prevalence of overweight in this study is higher than that obtained in other Nigerian sub-urban populations (18.5%^4^ and 20.3%^17^) but is within the range reported in urban populations (17.5%^13^ and 35.1%^18^). In addition, other studies in Nigerian populations showed much lower prevalence of obesity in sub-urban (13.1%^4^ and 12.5%^17^) and urban (9.8%^13^ and 8.2%^18^) populations than in this study. Although the setting for the current study is sub-urban, the town is very close to an urban commercial centre where most of the participants go for commercial and social activities. This proximate geographical location to an urban centre may account for the similar prevalence of overweight with other urban centres. Further, private motorised means of transport (notably motorcycle) are common in this population, which may foster low energy expenditure, and this may account for similar or higher prevalences of overweight and obesity as in urban populations. In addition, the dissimilarity in prevalence of overweight and obesity between the previous studies compared to the current study may be accounted for by the geographical location within Nigeria, the age range of participants, and setting of the study in terms of rural, sub-urban, and urban populations. The prevalences of overweight and obesity for women in this study falls within the ranges reported in studies from neighbouring African countries (27.1% to 50% for overweight and 20% to 37.1% for obesity), as does the prevalence of overweight for men (17.5% to 33%), whereas the prevalence of obesity for men in this study is much higher than the range in those studies (4.6% to 7%).^9^^,^^31^^–^^34^ However, prevalences of overweight and obesity among Kuwaiti (31.4% for overweight and 37.2% for obesity) and American (33.0% for overweight and 42.0% for obesity) adults is much higher than that observed in this study.^35^^,^^36^

Contrary to the finding in this study, a previous study in a Nigerian population reported the highest prevalence of obesity in the age range of 30–39 years.^13^ Similarly, Pasquet et al found that the prevalences of overweight and obesity increased from 20–29 years and peaked at 40–49 years in men and 50–59 years in women in a Cameroonian urban population, contrary to the finding in this study.^31^ These differences in the periods of the highest prevalence of obesity might be attributed to possible differences in socio-cultural characteristics and practices in those populations.^35^ The finding in this study might be partly attributed to decreased physical activity level in men and women and partly to hormonal changes in women that occur from about 45 years of age. These lifestyle and physiological phenomena may also explain the findings in this study of higher risk for overweight and obesity among individuals from the age of 41 years than those who are younger, and similar findings have been reported in previous studies.^13^^,^^14^^,^^33^^,^^34^^,^^37^

The finding of lower risk for overweight and obesity in males than in females is similar to findings in other studies in Nigerian populations.^13^^,^^14^ Similarly, the prevalence of obesity is about 1.5 to 2 times higher among women than men in countries with relatively low gross national product, such as Nigeria, whereas the prevalence of obesity is similar in men and women in developed countries.^38^ In fact, the findings for age and sex in association with body weight in this study and in previous similar studies suggest that age and sex are consistent predictors of body weight regardless of possible socio-cultural and genetic differences across populations.

The finding in our present study regarding distribution of obesity prevalence across occupational categories is in contrast with the report in a previous study on a Ghanaian population, in which participants with sedentary occupation had the highest prevalence of obesity.^32^ Our finding that prevalences of obesity were highest among those in skilled occupations, who have lesser engagement in manual labour, buttresses the fact that energy expenditure is a key factor in explaining body weight status. Similarly, individuals in skilled occupations, and not those in unskilled occupations, were found to present a higher risk for obesity than those in professional and unskilled occupations. This finding indicates that the risk for obesity is higher among those in skilled occupations who are likely to engage in less physically demanding activities than those in unskilled occupations. However, explaining the similar risks of obesity in professionals and unskilled individuals is quite difficult.

The highest prevalences of overweight and obesity were observed in participants who had only a primary education, and these findings are supported by the findings in other studies in Nigerian^13^^,^^18^ and other African populations.^31^^,^^32^ However, educational attainment seems not to be associated with BMI status.

Reviews of SES and obesity in developing countries have found a direct relationship between prevalence of obesity and increased SES,^39^^,^^40^ and this report only indicates an association between obesity and working in skilled occupations. These contrasting reports point to the possibility that pockets of populations exist in developing countries with obesity situations similar to those of high-income countries. According to Dinsa et al, the debate as to whether obesity primarily affects the rich or the poor continues in developing countries.^22^ Indeed, Monteiro et al noted that obesity was no longer a problem of only the higher socio-economic groups in developing countries.^21^ This, therefore, may call for caution in generalization of the socio-economic influence on overweight and obesity in low-income countries. Apart from the socio-economic influences, inherent psycho-social, cultural, and genetic factors may also be at play, which may confound the socio-economic influence on body weight. However, most studies reviewed which arrived at this generalization, including the current study, did not control for these possible factors.

In generalizing the findings in this study, certain limitations have to be considered. Although an overwhelming majority of the study population were of Christian faith, exclusion of the non-Christian minority may limit interpretation of the findings.^26^ Further, the participants were Christians who attended church services; this characteristic of the participants may limit extrapolation to all Christians in the population.^26^ In addition, considering that this study comprised a sample of Nigerian adults from a defined geographical area, the findings from it might not be easily generalized to the whole country. However, in a systematic review of studies on SES and obesity in developing countries, no major differences were found in the patterns of association between the national and sub-national data examined; this is true of the results of the present study, except for educational attainment. Although some limitations of BMI as a suitable method of determining body fatness have been recognized, such as failure to be able to differentiate individuals with pronounced muscular build, patterns observed in the studies that examined BMI concurrently with waist circumference and waist:hip ratio did not show any difference in patterns of obesity among people from developing countries.^22^

In conclusion, while the prevalence of overweight in this population is higher than the range in similar Nigerian settings, the prevalence of obesity is lower. The prevalences of overweight and obesity are within the ranges reported in neighbouring African populations. Further, the prevalence of overweight is higher in males than in females, but the reverse is the case for prevalence of obesity. While older age is associated with increased risk for overweight and obesity in this population, male sex is associated with reduced risk. However, while only work in skilled occupations is associated with obesity, tertiary educational attainment is associated with overweight.
